# What influences the use of professional home care for individuals with spinal cord injury? A cross-sectional study on family caregivers

**DOI:** 10.1038/s41393-019-0296-y

**Published:** 2019-05-24

**Authors:** Jianan Huang, Diana Pacheco Barzallo, Sara Rubinelli, Nadja Münzel, Mirjam Brach, Armin Gemperli

**Affiliations:** 1 Swiss Paraplegic Research, Nottwil, Switzerland; 2 Department of Health Sciences and Health Policy, University of Lucerne, Lucerne, Switzerland; 3 ParaHelp, Nottwil, Switzerland

**Keywords:** Health services, Public health

## Abstract

**Study Design:**

Cross-sectional survey.

**Objective:**

The objective of this study is to identify what characteristics of the family caregivers influenced the use of professional home care for persons with SCI in Switzerland.

**Setting:**

Community setting, nationwide in Switzerland.

**Methods:**

Questionnaires were filled out by the adult family caregivers of persons with SCI. Influence of characteristics of the caregivers was analyzed with regression models, adjusting for the characteristics of the person with SCI. Logistic regression was used for whether professional home care was used. Poisson regression was applied for the absolute and relative amount of professional home care.

**Results:**

In total, 717 family caregivers participated in the study (31% response rate). Among the participants, 33% hired professional home care for 10 h per week on average. The level of dependency of the persons with SCI had a significant influence on the utilization of care. The availability and proximity of the primary family caregiver, namely being spouse and cohabiting, reduced the amount of services used, whereas caregivers who worked full time employed more services. Higher levels of education and income increased the use of professional home care. Compared with their reference groups, caregivers with older age and those with a migratory background used comparable or larger absolute amount of professional services, which, however, represented a smaller proportion of total hours of care.

**Conclusions:**

Adequate support requires consideration of the characteristics of both the caregiver and of the person with SCI. The needs of family caregivers should also be assessed systematically in the needs assessment.

## Introduction

Persons with spinal cord injury (SCI), especially those with higher lesion levels, are dependent on care and assistance with daily living activities, with a majority provided by informal caregivers [1]. Caregiving carries a heavy objective and subjective burden [2, 3], which impacts the life of the informal caregiver [4]. The caregiving partners of persons with SCI reported a demand for external support [5]. However, low use of home-based services was observed in situations when family caregivers were involved [5, 6].

In previous SCI studies, the needs for caregiving of persons with SCI, indicated by the level of dependency, were found to be the major determinant of caregiving service [1, 7, 8]. The availability of informal care reduced the use of professional home care [7, 9]. Influence of caregiver characteristics were identified in studies of other care recipient populations. Older caregivers [10] and caregivers with higher levels of education [11] were more likely to seek support through professional home care. Despite the essential role of care provision by family members for persons with SCI, little was known about how the family caregiver’s characteristics influence utilization of professional home care [8].

In Switzerland, as an example of needs-based care, professional home care mainly provides medical care and assistance in activities of daily living (ADLs). The care is provided upon prescription from a doctor, according to the caregiving needs, which are assessed using standardized procedures by qualified service providers (Swiss Health Insurance Benefits Ordinance) [12]. Mandatory health or accident insurance covers the care service, for which private households pay a deducible and copayment [13], independent from their financial resources. However, assistance in instrumental ADLs (IADLs), such as housekeeping, requires self-payment.

The objective of this study was to identify what characteristics of the family caregivers influenced the use of professional home care for persons with SCI in Switzerland, specifically on (a) whether professional home care was used and (b) the amount of professional home care used. Implications for the family caregivers and service provision will be discussed based on the findings.

## Methods

### Data collection and variables

A questionnaire study was conducted in family caregivers of persons with SCI in Switzerland, from August 2016 to July 2017. Questionnaires were sent to persons with SCI, who forwarded it to their primary family caregivers. Eligible persons with SCI were contacted using information from the Swiss Spinal Cord Injury Cohort Study (SwiSCI) [14], cross-referenced with the membership database of the Swiss Paraplegic Association and patient databases of four SCI clinics in Switzerland.

The contacted persons with SCI fulfilled the inclusion criteria of SwiSCI study [14]: individuals with traumatic or non-traumatic SCI, over 16 years old, and permanently resided in Switzerland. Cases with injuries led by congenital conditions (e.g., spina bifida), with new SCI in palliative care, and with neurodegenerative conditions (e.g., multiple sclerosis, amyotrophic lateral sclerosis, and Guillan–Barré syndrome) were excluded. Individuals who were injured within the 2 years preceding the study were not invited, due to their insufficient experience with the use of health care and social services. Eligible participants consisted of family members over 18 years old, who assisted the person with SCI in ADL, e.g., washing and dressing, or IADL, e.g., shopping and housekeeping. Family caregivers who were unable to complete the questionnaire in German, French, or Italian were excluded.

In the questionnaire, family caregivers provided information about their socio-demographic characteristics, household members, employment, and satisfaction about own health (on a scale of 0–10). The questions included when they started the caregiving, their weekly time investment, activities in which they assisted, and who else also provided assistance. They further provided information regarding the person with SCI, including age, type of SCI, cause of SCI, and wheelchair dependency, which were used as indicators of caregiving needs. Whether they utilized professional home care and hours requested per week were also reported, which were treated as outcomes of the study.

### Statistical analysis

First, a binary indicator was applied as whether professional home care was used. This was fitted with a multiple logistic regression model, adjusting for characteristics of the caregiver and the person with SCI. The results are reported in odds ratio (OR), where a number higher than 1 indicates higher odds of using professional home care compared with the reference group.

Second, two Poisson regression models were used to analyze the amount requested for professional home care both in absolute and in relative terms. The relative term was defined as the proportion of professional home care with respect to the total hours of care (sum of professional home care and primary family caregiver), namely the weekly hours of professional home care divided by the total hours. It indicates the extent to which professional home care replaces the care undertaken by family caregivers. An increase in the absolute number of hours of professional home care may not always lead to an increase in the proportion. The results are reported as relative risk (RR), where a number higher than 1 indicates a higher quantity of professional home care compared with the reference group.

All statistical analyses were conducted with Stata version 14 for Windows (College Station, TX, USA). For all regression analyses, the missing values of independent variables were imputed with multiple imputation, using chained equation [15]. Estimates were reported out of a combined model with 20 imputed datasets, using Rubin’s rules [16]. Accounting for the multicollinearity, an analysis of variance inflated factors (VIF) was conducted. A maximum workload of 98 h per week (14 h a day) was assumed for the family members. Outliers exceeding this maximum, either in family or in professional home care, were replaced with 98 h (18 cases).

## Results

### Sample characteristics

In total, 717 questionnaires were returned, which resulted in a response rate of 31%. The family caregiver population was predominantly female (71%), with an average age of 57 years (Table 1). Most family caregivers were spouses (75%) and 83% of the participants cohabited with the care recipients. On average, they cared for the persons with SCI for the last 13 years and spent 21 h per week on caregiving tasks. One-third of the participants reported using professional home care (33%), for an average of 10 h per week.

**Table 1:** Characteristics of the sample

Characteristics	Total *N* = 717	Users of professional home care *N* = 238
Characteristics of the caregivers		
Sex, *n* (%)		
Male	203 (28.3)	65 (27.3)
Female	511 (71.3)	172 (72.3)
Age in years, *mean (SD)*	57.2 (13.9)	59.6 (14.4)
Migratory background, *n* (%)	149 (20.8)	55 (23.1)
Language region, *n* (%)		
German	526 (73.4)	166 (69.7)
French	154 (21.5)	57 (23.9)
Italian	30 (4.2)	12 (5.0)
Living abroad	7 (1.0)	3 (1.3)
Education level, *n* (%)		
No mandatory education	29 (4.0)	10 (4.2)
Mandatory school	178 (24.8)	59 (24.8)
Secondary II	314 (43.8)	108 (45.4)
Tertiary or higher	178 (24.8)	59 (24.8)
Personal monthly income, *n* (%)		
<1500 CHF	125 (17.4)	41 (17.2)
Between 1500 and 4500 CHF	332 (46.3)	106 (44.5)
Between 4500 and 7500 CHF	152 (21.2)	53 (22.3)
>7500 CHF	52 (7.3)	19 (8.0)
Missing	56 (7.8)	19 (8.0)
With children under 14 in household, *n* (%)	91 (12.7)	21 (8.8)
Living with the person with SCI, *n* (%)	596 (83.1)	193 (81.1)
Relationship to the person with SCI, *n* (%)		
Spouse/life partner	539 (75.2)	173 (72.7)
Child	39 (5.4)	19 (8.0)
Sibling	21 (2.9)	8 (3.4)
Mother/father	94 (13.1)	31 (13.0)
Other relative	8 (1.1)	3 (1.3)
Satisfaction with own health, *n* (%)		
Low (0–5)	141 (19.7)	59 (24.8)
Medium (6–8)	320 (44.6)	111 (46.6)
High (9–10)	242 (33.8)	65 (27.3)
Employment, *n* (%)		
Not in employment	327 (45.6)	123 (51.7)
Employed in part-time	275 (38.4)	80 (33.6)
Employed in full-time	97 (13.5)	32 (13.4)
Time since caregiving in years, *mean (SD)*	12.9 (11.5)	10.9 (9.9)
Hours/week of care by family caregiver, *mean (SD)*	21.1 (25.8)	29.8 (31.7)
Other informal caregiver(s) involved, *n* (%)	291 (40.6)	92 (38.7)
Professional home care utilized, *n* (%)	238 (33.2)	-
Hours/week of professional home care, *mean (SD)*	-	10.1 (9.9)
GP as primary contact for health problem, *n* (%)	493 (68.8)	161 (67.6)
In need of more professional home care, *n* (%)		
No, satisfied with current situation	518 (72.2)	155 (65.1)
Yes, in need of more service	161 (22.5)	80 (33.6)
Missing	38 (5.3)	3 (1.3)
Characteristics of the persons with SCI		
Sex, *n* (%)		
Male	519 (72.4)	169 (71.0)
Female	183 (25.5)	68 (28.6)
Age in years, *mean (SD)*	56.4 (16.3)	59.6 (16.9)
Cause of SCI		
Due to an accident	509 (71.0)	167 (70.2)
Due to disease	125 (17.4)	47 (19.7)
Other cause	58 (8.1)	23 (9.7)
Type of SCI, *n* (%)		
Paraplegic	432 (60.3)	110 (46.2)
Tetraplegic	225 (31.4)	124 (52.1)
Missing	60 (8.4)	-
Wheelchair dependency, *n* (%)		
Completely dependent on wheelchair	484 (67.5)	203 (85.3)
Able to stand	23 (3.2)	8 (3.4)
Partially able to walk	169 (23.6)	23 (9.7)
Missing	41 (5.7)	-
Time since injury in years, *mean (SD)*	17.5 (14.3)	15.2 (13.5)

*CHF* Swiss Franc, *GP* general practitioner, *SCI* spinal cord injury

The numbers of missing values are <5% if not specified otherwise.

Persons with SCI, who received care from family members, were predominantly male (72%) and 56 years old, on average. Over half were paraplegic (60%) and the injury was caused by an accident in 71% of the cases. Most were wheelchair dependent (68%) and had lived with the injury for 18 years, on average.

### Use of professional home care

Table 2 details the results of the logistic regression on whether professional home care was used, predicted by the characteristics of the family caregiver and the person with SCI. In general, the use of professional home care is unrelated to the characteristics of the family caregiver, except the relationship to the care recipient and caregiver’s satisfaction with own health. Family members who were not spouses were more likely to use professional home care (OR 1.92, 95% confidence interval (CI) 1.01 to 3.66). The family caregivers who are highly satisfied with their own health status were less likely to use professional home care (OR 0.56, 95% CI 0.32 to 0.98 vs. those who reported low satisfaction).

**Table 2:** Logistic regression: association of professional home care utilization with characteristics of the caregiver and of the person with SCI

Utilization of professional home care	Odds ratio (95% CI)
Characteristics of the caregivers	
Female caregiver	1.47 (0.82–2.63)
Age	
≤40 years old	Reference
41–60 years old	0.65 (0.34–1.26)
61–70 years old	0.86 (0.38–1.93)
Above 70 years old	0.65 (0.25–1.72)
Migratory background	1.33 (0.80–2.22)
Language region	
German	Reference
French	1.32 (0.83–2.08)
Italian	1.44 (0.57–3.66)
Living abroad	1.26 (0.20–7.94)
Education level	
No mandatory school	Reference
Mandatory school	1.25 (0.44–3.53)
Secondary II	1.51 (0.55–4.13)
Tertiary or higher	1.23 (0.42–3.62)
Personal monthly income	
<1500 CHF	Reference
Between 1500 and 4500 CHF	0.80 (0.46–1.37)
Between 4500 and 7500 CHF	1.15 (0.58–2.30)
>7500 CHF	1.84 (0.69–4.91)
With children under 14 in household	0.77 (0.39–1.51)
Living with the person with SCI	0.95 (0.49–1.87)
Relationship to the person with SCI	
Spouse/life partner	Reference
Others family members	1.92 (1.01–3.66)*
Satisfaction with own health	
Low (0–5)	Reference
Medium (6–8)	0.72 (0.43–1.19)
High (9–10)	0.56 (0.32–0.98)*
Employment	
Not in employment	Reference
Employed in part-time	0.93 (0.55–1.57)
Employed in full-time	1.10 (0.53–2.30)
Time since caregiving	
≤15 years	Reference
>15 years	0.66 (0.35–1.27)
Other informal caregivers involved	0.99 (0.66–1.50)
GP as primary contact for health problem	0.86 (0.56–1.31)
Characteristics of the persons with SCI	
Person with SCI being female	1.69 (0.96–2.96)
Age	
≤40 years old	Reference
41–60 years old	1.60 (0.86–2.99)
61–70 years old	3.71 (1.77–7.77)***
>70 years old	8.04 (3.57–18.10)***
Cause of SCI	
Due to an accident	Reference
Due to disease	1.89 (1.10–3.25)*
Other cause	1.68 (0.80–3.53)
Type of SCI	
Paraplegic	Reference
Tetraplegic	5.51 (3.60–8.44)***
Wheelchair dependency	
Completely dependent on wheelchair	Reference
Able to stand	0.36 (0.12–1.08)
Partially able to walk	0.09 (0.05–0.16)***
Time since injury	
≤15 years	Reference
>15 years	0.57 (0.31–1.03)

Odds ratios > 1 indicate higher odds of hiring professional home care. Pseudo-*R*^2^ of the respective imputed datasets ranged from 24% to 25%

Odds ratio based on multiple logistic regression, adjusting for all variables shown in the table

*CHF*  Swiss Francs, *CI* confidence interval, *GP* general practitioner, *SCI* spinal cord injury

*p < 0.05, **p < 0.01, ***p < 0.001

The use of professional home care is significantly associated with characteristics of the persons with SCI. Older persons with SCI (OR 3.71, 95% CI 1.77 to 7.77 in persons aged 61–70 years and OR 8.04, 95% CI 3.57 to 18.10 in persons older than 70 years, vs. those under 40 years old) and persons with tetraplegia (OR 5.51, 95% CI 3.60 to 8.44) were more likely to employ professional home care. Also, persons whose SCI was related to disease were more likely to use professional home care (OR 1.89, 95% CI 1.10 to 3.25 vs. due to an accident). Persons with SCI who were partially able to walk were less likely to use professional home care when compared with wheelchair-dependent persons (OR 0.09, 95% CI 0.05 to 0.16 vs. those dependent on a wheelchair).

### Amount of professional home care

#### Absolute hours of professional home care

The number of hours requested for professional home care was associated with several characteristics of the family caregiver (Table 3). More hours of professional home care were requested by female caregivers (RR 1.56, 95% CI 1.33 to 1.84), caregivers with a migratory background (RR 1.32, 95% CI 1.15 to 1.50), caregivers residing in the French-speaking part of Switzerland (RR 1.57, 95% CI 1.41 to 1.74 vs. German-speaking), caregivers with higher education level (RR 1.48, 95% CI 1.01 to 2.17 in mandatory education, RR 1.91, 95% CI 1.31 to 2.77 in secondary II and RR 1.95, 95% CI 1.34 to 2.84 in tertiary or higher vs. no mandatory education), and caregivers with monthly income above 7500 CHF (RR 1.36, 95% CI 1.04 to 1.77 vs. <1500 CHF). Also, more hours were hired when the primary caregiver was a non-spousal relative (RR 1.38, 95% CI 1.17 to 1.63). The same result holds for cases where other informal caregivers were involved (RR 1.31, 95% CI 1.18 to 1.46). Fewer hours of professional home care were requested when the caregiver’s income was between 1500 and 4500 CHF (RR 0.85, 95% CI 0.73 to 1.00 compared with <1500 CHF), when the caregiver lived with the person with SCI (RR 0.78, 95% CI 0.66 to 0.92), when the caregiver reported a high satisfaction with their own health (RR 0.74, 95% CI 0.61 to 0.89 vs. those with low satisfaction), and those who indicated the general practitioner (GP) as primary contact for health problems (RR 0.84, 95% CI 0.76 to 0.94).

**Table 3:** Poisson regression: association of utilized hours of service with characteristics of the caregivers and of the persons with SCI

Hours of professional home care service	Relative risk in absolute hours of professional home care (95% CI)	Relative risk in proportion of professional home care (95% CI)
Characteristics of the caregivers		
Female caregiver	1.56 (1.33–1.84)***	1.63 (1.29–2.06)*
Age		
≤40 years old	Reference	Reference
41–60 years old	0.95 (0.81–1.12)	0.78 (0.66–0.92)**
61–70 years old	0.88 (0.72–1.06)	0.73 (0.59–0.89)**
>70 years old	1.13 (0.89–1.44)	0.63 (0.49–0.81)*
Migratory background	1.32 (1.15–1.50)***	0.84 (0.75–0.95)**
Language region		
German	Reference	Reference
French	1.57 (1.41–1.74)***	1.41 (1.27–1.58)*
Italian	1.26 (0.98–1.63)	1.16 (0.91–1.48)
Living abroad	0.80 (0.50–1.29)	0.94 (0.58–1.52)
Education level		
No mandatory school	Reference	Reference
Mandatory school	1.48 (1.01–2.17)*	1.28 (0.91–1.81)
Secondary II	1.91 (1.31–2.77)***	1.75 (1.25–2.44)**
Tertiary or higher	1.95 (1.34–2.84)***	1.76 (1.24–2.51)**
Personal monthly income		
<1500 CHF	Reference	Reference
Between 1500 and 4500 CHF	0.85 (0.73–1.00)*	1.06 (0.89–1.26)
Between 4500 and 7500 CHF	1.12 (0.91–1.37)	1.35 (1.11–1.65)**
>7500 CHF	1.36 (1.04–1.77)*	1.72 (1.30–2.27)***
With children under 14 in household	1.02 (0.87–1.20)	1.15 (0.97–1.36)
Living with the person with SCI	0.78 (0.66–0.92)**	0.54 (0.46–0.63)***
Relationship to the person with SCI		
Spouse/life partner	Reference	Reference
Others family members	1.38 (1.17–1.63)***	1.87 (1.59–2.20)***
Satisfaction with own health		
Low (0–5)	Reference	Reference
Medium (6–8)	0.95 (0.83–1.09)	0.88 (0.78–1.00)*
High (9–10)	0.74 (0.61–0.89)**	0.76 (0.65–0.90)**
Employment		
Not in employment	Reference	
Employed in part-time	0.90 (0.78–1.04)	0.93 (0.81–1.07)
Employed in full-time	1.18 (0.97–1.44)	1.25 (1.02–1.55)*
Time since caregiving		
≤15 years	Reference	Reference
>15 years	0.86 (0.63–1.18)	0.85 (0.65–1.11)
Other informal caregivers involved	1.31 (1.18–1.46)***	1.44 (1.29–1.59)***
GP as primary contact for health problem	0.84 (0.76–0.94)**	0.77 (0.68–0.87)***
Characteristics of the persons with SCI		
Person with SCI being female	1.53 (1.31–1.79)***	1.52 (1.26–1.84)***
Age		
≤40 years old	Reference	Reference
41–60 years old	1.17 (1.00–1.38)	1.36 (1.16–1.59)***
61–70 years old	2.02 (1.68–2.42)***	2.10 (1.75–2.52)***
>70 years old	3.03 (2.48–3.70)***	2.96 (2.39–3.66)***
Cause of SCI		
Due to an accident	Reference	Reference
Due to disease	1.44 (1.23–1.69)***	1.29 (1.12–1.48)***
Other cause	1.39 (1.18–1.64)***	1.17 (0.98–1.39)
Type of SCI		
Paraplegic	Reference	Reference
Tetraplegic	3.10 (2.78–3.45)***	1.76 (1.58–1.96)***
Wheelchair dependency		
Completely dependent on wheelchair	Reference	Reference
Able to stand	0.29 (0.19–0.43)***	0.27 (0.18–0.40)***
Partially able to walk	0.13 (0.10–0.16)***	0.27 (0.22–0.33)***
Time since injury		
≤15 years	Reference	Reference
>15 years	0.49 (0.37–0.64)***	0.63 (0.51–0.78)***

Relative risks > 1 indicate higher amount of professional home care.

Relative risks in absolute hours: number of hours of professional home care as outcome. Pseudo-*R*^2^ of respective datasets ranged from 33% to 34%

Relative risks in proportion: proportion of hours of professional home care among the total hours of care (hours of family members and professional home care combined) as outcome. Pseudo-*R*^2^ of respective datasets ranged from 29% to 30%

Relative risk based on Poisson regression, adjusting for all variables shown in the table

*CHF* Swiss Francs, *CI* confidence interval, *GP* general practitioner, *SCI* spinal cord injury

*p < 0.05, **p < 0.01, ***p < 0.001

More hours of services were requested for women with SCI (RR 1.53, 95% CI 1.31 to 1.79), older care recipients (RR 2.02, 95% CI 1.68 to 2.42 in persons aged 61–70 years, RR 3.03, 95% CI 2.48 to 3.70 in persons above 70 years old, vs. those under 40 years old), persons whose injury was caused by reasons other than an accident (RR 1.44, 95% CI 1.23 to 1.69 in disease-induced injury and RR 1.39, 95% CI 1.18 to 1.64 in others vs. due to an accident), and persons with tetraplegia (RR 3.10, 95% CI 2.78 to 3.45). Fewer hours of professional home care were requested for persons with SCI, who were not wheelchair dependent (RR 0.29, 95% CI 0.19 to 0.43 in those who were able to stand and RR 0.13, 95% CI 0.10 to 0.16 in those who were able to walk compared with the wheelchair dependent ones) and for those who had lived with the injury for over 15 years (RR 0.49, 95% CI 0.37 to 0.64).

#### Proportion of professional home care

Out of the total care provision, the proportion of professional home care was greater among female caregivers (RR 1.63, 95% CI 1.29 to 2.06), caregivers residing in the French-speaking part of Switzerland (RR 1.41, 95% CI 1.27 to 1.58 vs. German-speaking), caregivers with higher education levels (RR 1.75, 95% CI 1.25 to 2.44 in secondary II and RR 1.76, 95% CI 1.24 to 2.51 compared with no mandatory education), caregivers with a higher income level (RR 1.35, 95% CI 1.11 to 1.65 in 4500–7500 CHF and RR 1.72, 95% CI 1.30 to 2.27 in above 7500 CHF compared with <1500 CHF), non-spousal caregivers (RR 1.87, 95% CI 1.59 to 2.20), and caregivers who were employed full time (RR 1.25, 95% CI 1.02 to 1.55 compared with those not employed), and in cases where other informal caregivers were involved (RR 1.44, 95% CI 1.29 to 1.59). The proportion was smaller for family caregivers living with the person with SCI (RR 0.54, 95% CI 0.46 to 0.63), for caregivers who reported higher levels of satisfaction with their own health (RR 0.88, 95% CI 0.78 to 1.00 in medium satisfaction and RR 0.76, 95% CI 0.65 to 0.90 in high satisfaction, vs. those with low satisfaction), and for those who indicated GP as their primary contact (RR 0.77, 95% CI 0.68 to 0.87).

Although the family caregiver’s age was unrelated to the hours of professional home care requested, the proportion of professional home care decreased with the age of the family caregiver (RR 0.78, 95% CI 0.66 to 0.92 in 41–60-year-olds, RR 0.73, 95% CI 0.59 to 0.89 in 61–70-year-olds, and RR 0.63, 95% CI 0.49 to 0.81 in those older than 70 years, vs. those under 40 years old). Caregivers with a migratory background, despite using more professional home-care hours, used a significantly smaller proportion of professional home care when compared with Swiss-born caregivers (RR 0.84, 95% CI 0.75 to 0.95).

A larger proportion of professional home care was requested for female care recipients (RR 1.52, 95% CI 1.26 to 1.84), persons with SCI who were older (RR 1.36, 95% CI 1.16 to 1.59 in persons aged 41–60 years, RR 2.10, 95% CI 1.75 to 2.52 in persons aged 61–70 years, and RR 2.96, 95% CI 2.39 to 3.66 in persons above 70 years old), had tetraplegia (RR 1.76, 95% CI 1.58 to 1.96), whose injury was caused by disease (RR 1.29, 95% CI 1.12 to 1.48 vs. due to accident), and persons that were wheelchair dependent (RR 0.27, 95% CI 0.18 to 0.40 in persons able to stand, and RR 0.27, 95% CI 0.22 to 0.33 in persons partly able to walk compared with the wheelchair dependent). Persons with SCI, who lived with the injury for over 15 years, requested a smaller proportion of professional home care (RR 0.63, 95% CI 0.51 to 0.78).

The results did not show severe multicollinearity (Supplementary Table 1). Most of the predictors had VIF <5. The only exception was education of the family caregiver (VIF ranged 6.12–8.08 across the categories).

## Discussion

Although the characteristics of the persons with SCI were decisive for the use of professional home care, the amount of the services used was linked to characteristics of the family caregivers. As for the persons with SCI, those with older age, tetraplegia, and SCI caused by a disease tend to develop more health conditions [17] and thus employed more professional home care. A higher use of professional home care was found in persons with SCI, who were dependent on wheelchairs due to their physical limitations. These findings are in line with similar studies done in the United States [9, 18], in which higher level of injury, dependency, and older age of the person with SCI increased the use of home care services. In contrast, persons with SCI, who had lived with their injury for over 15 years employed less professional home care. This could reflect an increased ability to handle daily activities independently over time [1]. The results underline the needs-based care provision in Switzerland, which centers the care demand of the person with SCI.

Apart from the care demand in focus, greater availability and proximity of the primary family caregiver reduced the amount of professional home care requested. Married persons with SCI received substantial care from their spouses and, thus, reported less use of professional home care [7, 9]. Spouses and cohabiting relatives still seemed to be the first source of caregiving. They may need to ensure their availability to provide assistance and care. When family caregivers were employed full time, however, they compensated larger part of the caregiving with professional services, because their availability for caring was limited.

Contrary to a previous study [19], the involvement of additional informal caregivers (family and friends) in our study increased the use of professional home care. This may be due to the fact that the situation overall is particularly demanding. The primary caregiver then needs both informal and formal support to manage the demand.

Adding to the existing research on SCI [8], this study found a significant relationship between socioeconomic characteristics of a caregiver and the amount of professional home care used, either in absolute or relative term. Education facilitates the use of home-based care as more highly educated caregivers are more able to navigate through the healthcare system [11]. In addition, caregivers with income above 4500 CHF replaced their caregiving with more professional home care. This might reflect that households with more financial resources are less dependent on public coverage for medical expenses and more flexible with the copayment [20]. As noted, support in household tasks relies on family caregivers or additional financial resources in Switzerland. The caregivers with more capacity could thus benefit more from the support system.

Contrary to our expectation, older family caregivers used comparable amount of professional home care as the younger caregivers and those with a migratory background hired more professional home care than Swiss-born caregivers; yet, in both cases the proportion of professional home care decreased. This finding suggests that these groups provided more caregiving, while also obtaining high levels of professional support. There are two potential reasons for this. First, the amount of professional home care does not sufficiently cover the actual need for care, requiring family caregivers to invest more time. Second, a majority of the caregiver’s assistance may not be related to care and may not be covered by insurance. For example, older caregivers may need more support for housekeeping tasks [10] and the self-payment becomes a barrier. As age has an adverse relation with the family caregiver’s quality of life [21], the older caregivers are more affected by the caregiving. In families with a migratory background, family members play an even more substantial role in caregiving [22]. These caregivers may take over more responsibilities than their Swiss-born peers, thereby decreasing the proportion of professional support in the total provision of care. As found in the United States, Latino caregivers reported smaller social support networks and less help-seeking coping behavior than their European American peers [23]. The current needs assessment tool only entails simple information about the caregivers: the possibility of discontinuing due to burden and estimated time investment in the activities that they assisted (ADL/medication management/nursing) [24]. It will be too late to provide support or to adjust the care provision by the time when a family caregiver reports the burden and discontinues the caregiving.

### Limitations

This study was conducted with three limitations in mind. First, the questionnaires were forwarded to the primary family caregivers by the persons with SCI. This may partly explain the low response rate. Family members might not be considered as caregivers if they are responsible for usual household tasks. Therefore, the study may have presented family members who were more involved in assistance of ADL. Furthermore, time investment by other informal caregivers was not captured. Data analysis was based only on the time investment reported by the primary caregiver. This limits the accuracy of the calculation for the proportion of professional home care. The proportion of professional home care might have been smaller in cases with other informal caregivers.

Second, the questionnaire was provided in the three official languages in Switzerland. Caregivers who did not understand any of these languages were excluded from participating. Almost 10% of the first-generation immigrants in Switzerland were estimated to lack proficiency of any of these three official languages [25]. Given the findings of this study, further research is needed for caregivers with migratory backgrounds.

Third, the study lacks the consideration of sociocultural factors on community level. The increased use of service in French-speaking region can be traced back to either the cultural diversity or organizational difference, which can influence utilization of health care [8, 26]. Research is thus needed to explore the influence of culture, attitude toward professional home care, and service delivery.

## Conclusion

Utilization of professional home care differed across characteristics of the family caregivers. Planning of adequate support requires consideration of the characteristics of both the caregiver and the individual with SCI, reflecting on how the care provision can account for them, such as the age and migratory background of the family caregiver. Otherwise, a dropout may occur due to escalating needs, which leads to unexpected deficits in caregiving. The needs of family caregivers should hence be assessed systematically in the regular needs assessment.

### Data archiving

The datasets generated and analyzed in the current study are available from the corresponding author on request.

## Supplementary information

Supplementary Table 1

## References

[1] Smith EM, Boucher N, Miller WC (2016). Caregiving services in spinal cord injury: a systematic review of the literature. Spinal Cord.

[2] Scholten EWM, Kieftenbelt A, Hillebregt CF, de Groot S, Ketelaar M, Visser-Meily JMA (2018). Provided support, caregiver burden and well-being in partners of persons with spinal cord injury 5 years after discharge from first inpatient rehabilitation. Spinal Cord.

[3] Weitzenkamp DA, Gerhart KA, Charlifue SW, Whiteneck GG, Savic G (1997). Spouses of spinal cord injury survivors: the added impact of caregiving. Arch Phys Med Rehabil.

[4] Gordon JR, Pruchno RA, Wilson-Genderson M, Murphy WM, Rose M (2011). Balancing caregiving and work. J Fam Issues.

[5] Post MW, Bloemen J, de Witte LP (2005). Burden of support for partners of persons with spinal cord injuries. Spinal Cord.

[6] Tough H, Brinkhof MW, Siegrist J, Fekete C (2017). Subjective caregiver burden and caregiver satisfaction: the role of partner relationship quality and reciprocity. Arch Phys Med Rehabil.

[7] Walker EA, Cao Y, Edles PA, Acuna J, Sligh-Conway C, Krause JS (2015). Racial-ethnic variations in paid and unpaid caregiving: findings among persons with traumatic spinal cord injury. Disabil Health J.

[8] Guilcher SJ, Craven BC, McColl MA, Lemieux-Charles L, Casciaro T, Jaglal SB (2012). Application of the Andersen’s health care utilization framework to secondary complications of spinal cord injury: a scoping review. Disabil Rehabil.

[9] Weitzenkamp DA, Whiteneck GG, Lammertse DP (2002). Predictors of personal care assistance for people with spinal cord injury. Arch Phys Med Rehabil.

[10] Levesque L, Cossette S, Potvin L, Benigeri M (2000). Community services and caregivers of a demented relative: users and those perceiving a barrier to their use. Can J Aging.

[11] van Houtven CH, Norton EC (2004). Informal care and health care use of older adults. J Health Econ.

[12] Swiss Federal Department of Home Affairs. Verordnung des EDI über Leistungen in der obligatorischen Krankenpflegeversicherung: SR 832.112.31 (Swiss Health Insurance Benefits Ordinance: SR 832.112.31) 1995. Accessed on 19 Mar 2019. Available from: https://www.admin.ch/opc/de/classified-compilation/19950275/index.html.

[13] Weaver F. Long-term care financing in Switzerland. In: Costa-Font J, Courbage C (eds). Financing long-term care in Europe: institutions, markets and models. Palgrave Macmillan, Basingstoke, UK; 2012, p. 279–99.

[14] Post MW, Brinkhof MW, von Elm E, Boldt C, Brach M, Fekete C (2011). Design of the Swiss Spinal Cord Injury Cohort Study. Am J Phys Med Rehabil.

[15] van Buuren S (2007). Multiple imputation of discrete and continuous data by fully conditional specification. Stat Methods Med Res.

[16] Royston P (2004). Multiple imputation of missing values. Stata J.

[17] Brinkhof MW, Al-Khodairy A, Eriks-Hoogland I, Fekete C, Hinrichs T, Hund-Georgiadis M (2016). Health conditions in people with spinal cord injury: contemporary evidence from a population-based community survey in Switzerland. J Rehabil Med.

[18] Robinson-Whelen S, Rintala DH (2003). Informal care providers for veterans with SCI: who are they and how are they doing?. J Rehabil Res Dev.

[19] Toseland RW, McCallion P, Gerber T, Banks S (2002). Predictors of health and human services use by persons with dementia and their family caregivers. Soc Sci Med.

[20] Tokunaga M, Hashimoto H, Tamiya N (2015). A gap in formal long-term care use related to characteristics of caregivers and households, under the public universal system in Japan: 2001–10. Health Policy.

[21] McCullagh E, Brigstocke G, Donaldson N, Kalra L (2005). Determinants of caregiving burden and quality of life in caregivers of stroke patients. Stroke.

[22] Kohn J, Tov E, Hanetseder C, Hungerbühler H. Pflegearrangements und Einstellung zur Spitex bei Migrantinnen und Migranten in der Schweiz. Eine Studie im Auftrag des Nationalen Forums Alter und Migration (Caregiving arrangement and attitude towards professional home care among immigrants in Switzerland. A study by order of the National Forum of Aging and Migration). Basel/Bern: FHNW/SRK: Swiss Federal Office of Public Health; 2013.

[23] Valle R, Yamada AM, Barrio C (2004). Ethnic differences in social network help-seeking strategies among Latino and Euro-American dementia caregivers. Aging Ment Health.

[24] Anliker M, Bartelt G, DuPasquier J, Gilgen R, Müller P, Staudenmaier B. Handbuch RAI-Home-Care Schweiz (Handbook of RAI-Home Care Switzerland), translated, tested in pilot and revised from RAI-Home Care Assessment Manual for version 2.0, 1999. St. Gallen: Q-Sys AG in collaboration with Swiss Association of Spitex (Professional Home Care); 2009.

[25] Swiss Federal Statistical Office. Personen, die 3,2,1 oder keine Landessprache beherrschen (Persons, who master 3,2,1 or none of the Swiss official language) Neuchâtel 2015. Accessed on 19 Mar 2019. Available from: https://www.bfs.admin.ch/bfs/de/home/statistiken/bevoelkerung/migration-integration/integrationindikatoren/alle-indikatoren/sprache/3-2-1-0-landessprachen.assetdetail.5546599.html.

[26] Derose KP, Varda DM (2009). Social capital and health care access: a systematic review. Med Care Res Rev.

