# Supplementary material for: What influences the use of professional home care for individuals with spinal cord injury? A cross-sectional study on family caregivers
Source: Spinal Cord. 2019 May 24;57(11):924–32. doi: 10.1038/s41393-019-0296-y (PMC6892416; doi:10.1038/s41393-019-0296-y)
Supplement: Supplementary file 1 — Supplementary Table 1 [file 41393_2019_296_MOESM1_ESM.pdf]

Supplementary Table 1 Variance inflation factors (VIF) of the variables

Predictors*	VIF
**Characteristics of the caregivers**	
Female caregiver	2.37
Age	
41 – 60 years old	3.09
61 – 70 years old	4.17
Above 70 years old	4.24
Migratory background	1.31
Language region	
French	1.07
Italian	1.11
Living abroad	1.13
Education	
Mandatory school (Secondary I)	6.12
Secondary II	8.08
Tertiary or higher	7.16
Personal monthly income	
Between 1500 and 4500 CHF	2.23
Between 4500 and 7500 CHF	2.41
Above 7500 CHF	2.10
With children under 14 in household	1.31
Living with the SCI person	2.01
Other relatives of the person with SCI as caregiver	
Others family members	2.40
Satisfaction about health	
Medium (6 – 8)	2.12
High (9 – 10)	2.28
Employment	
Employed in part-time	2.17
Employed in full-time	2.13
Other informal caregivers involved	1.18
Time since caregiving > 15 years	
More than 15 years	2.59
GP as primary contact for health problem	1.11
Characteristics of the persons with SCI	
Person with SCI being female	2.11
Age	
41 – 60 years old	2.44
61 – 70 years old	2.96
Above 70 years old	3.07
Tetraplegic	1.11
Cause of SCI	
Due to disease	1.23
Other cause	1.18
Wheelchair dependency	
Able to stand	1.08
Partially able to walk	1.17
Time since Injury > 15 years	2.51
**Mean VIF**	**2.49**

*The reference categories were omitted.
